# CCfrag: scanning folding potential of coiled-coil fragments with AlphaFold

**DOI:** 10.1093/bioadv/vbae195

**Published:** 2024-12-06

**Authors:** Mikel Martinez-Goikoetxea

**Affiliations:** Department of Protein Evolution, Max Planck Institute for Biology, Tübingen 72076, Germany

## Abstract

**Motivation:**

Coiled coils are a widespread structural motif consisting of multiple α-helices that wind around a central axis to bury their hydrophobic core. While AlphaFold has emerged as an effective coiled-coil modeling tool, capable of accurately predicting changes in periodicity and core geometry along coiled-coil stalks, it is not without limitations, such as the generation of spuriously bent models and the inability to effectively model globally non-canonical-coiled coils. To overcome these limitations, we investigated whether dividing full-length sequences into fragments would result in better models.

**Results:**

We developed CCfrag to leverage AlphaFold for the piece-wise modeling of coiled coils. The user can create a specification, defined by window size, length of overlap, and oligomerization state, and the program produces the files necessary to run AlphaFold predictions. The structural models and their scores are then integrated into a rich per-residue representation defined by sequence- or structure-based features. Our results suggest that removing coiled-coil sequences from their native context can improve prediction confidence and results in better models. In this article, we present various use cases of CCfrag and propose that fragment-based prediction is useful for understanding the properties of long, fibrous coiled coils by revealing local features not seen in full-length models.

**Availability and implementation:**

The program is implemented as a Python module. The code and its documentation are available at https://github.com/Mikel-MG/CCfrag.

## 1 Introduction

Coiled coils consist of multiple α-helices that wind around a central axis to bury their hydrophobic core. They are widespread in proteomes, where they can be found in a variety of forms, depending on the number and orientation of their constituent helices (Lupas and Bassler 2017). This topological diversity is underpinned by the seven-residue *heptad* repeat, labeled *a*–*g*, where the *a* and *d* positions form the core. The geometry of coiled-coil interaction, known as knobs-into-holes, involves the core residue of a helix (knob) packing into a cavity formed by four residues (hole) of an adjacent helix (Crick 1953a). They are the best understood protein fold, as indicated by the number of programs that are able to detect coiled-coil forming propensity from sequence (Lupas *et al.* 2017), and by the existence of the Crick parametric equations that describe their backbone (Crick 1953b), which have been implemented in programs capable of generating idealized coiled-coil atomic models (Offer *et al.* 2002, Grigoryan and DeGrado 2011, Wood *et al.* 2017). In spite of this, coiled-coil structural prediction has remained a substantial challenge, due to the fact that, although preponderantly repetitive in sequence and structure, coiled-coil domains are rarely without occasional interruptions in the form of non-heptad repeats, which locally alter their packing interactions and geometry. Although alternative approaches to deal with these local interruptions have also been developed, in the form of fragment-based routines (Guzenko and Strelkov 2018), these are limited to certain oligomeric states and tend to enforce coiled-coil structure in their predicted models.

Even as a general-purpose protein structure model, AlphaFold (Evans *et al.* 2022, Jumper *et al.* 2021) has demonstrated an outstanding ability to accurately model coiled-coil domains, particularly with respect to their supercoiling and core geometry (Madaj *et al.* 2024). This can be appreciated in a recent large-scale survey into the homo-oligomerization landscape across domains of life, which showed coiled-coil interfaces to be widespread across proteomes (Schweke *et al.* 2024). Additionally, it has been shown that it can be used to inform of dynamic protein conformations (Wayment-Steele *et al.* 2024, Winski *et al.* 2024). Despite its merits, AlphaFold cannot robustly model some long coiled coils, for which it often outputs oddly bent models that feature spurious contacts or even atomic clashes. We have also observed that it does not generate confident models for globally non-canonical coiled coils, which are modeled with poor or absent side-chain packing (Martinez-Goikoetxea and Lupas 2023). We hypothesize that these issues are related to the poor representation of these kinds of structures in their full-length form in the Protein Data Bank, from which AlphaFold was trained. It is possible that this introduces a bias in the AlphaFold model generation, which would tend to increase the number of contacts in long fibrous proteins by spuriously bending them.

Motivated by these observations, we wondered whether computing AlphaFold models of short overlapping windows along a sequence would yield better quality models by simplifying the prediction task. Thus, we developed CCfrag, a pipeline to automate the division of a sequence into fragments and the subsequent integration of the corresponding AlphaFold models into a rich per-residue representation. Our results suggest that not only does this improve the modeling quality of challenging coiled coils, but additionally, it can be used to scan sequences for local structural properties not observed in full-length coiled-coil models. We anticipate that this framework will be of particular interest in the context of understanding long fibrous coiled coils, such as myosins and kinesins.

## 2 Methods

**Figure 1.**
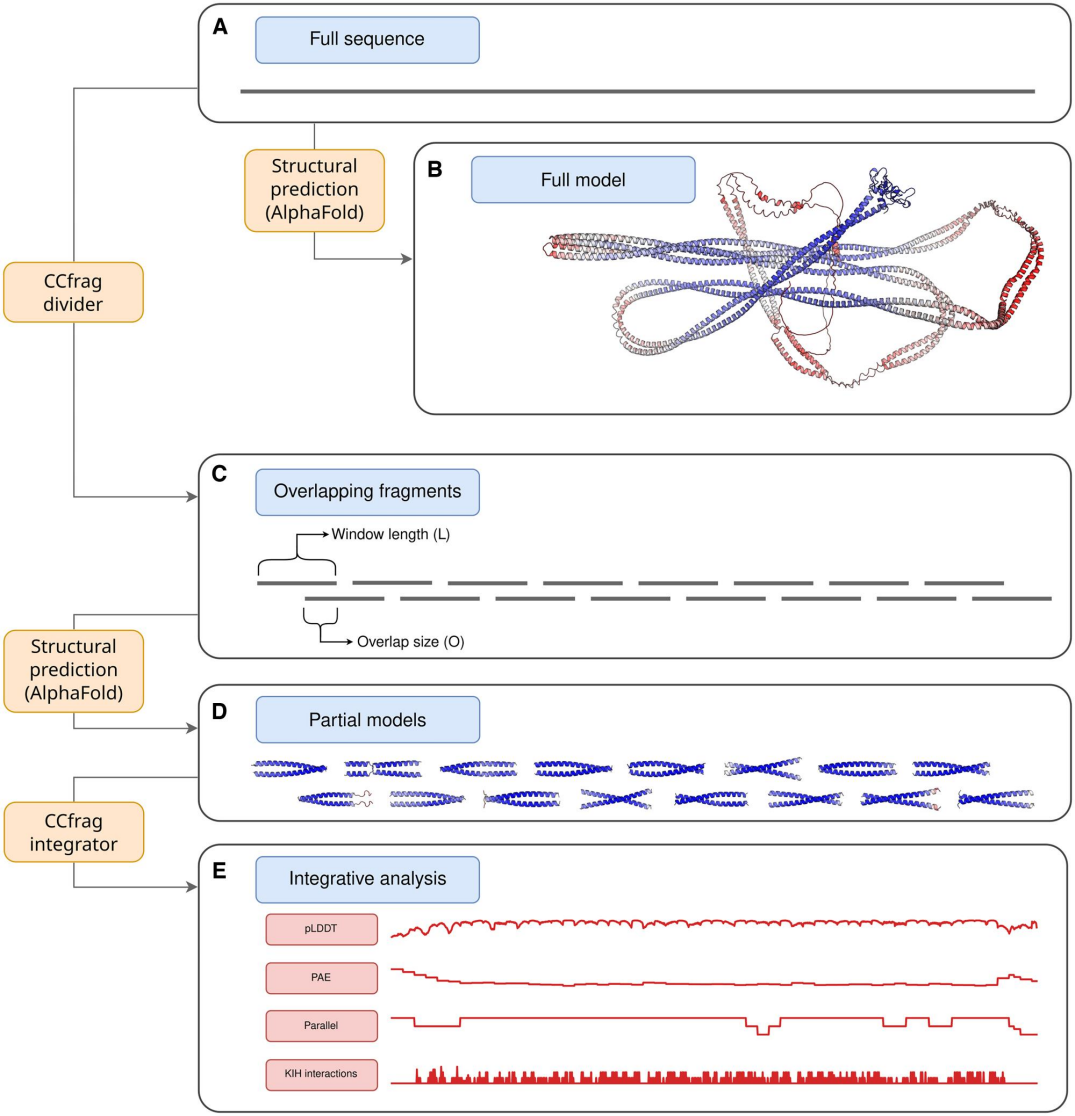
Schematic representation of the CCfrag pipeline. Modeling long coiled-coil domains with AlphaFold generally yields suboptimal models; (A, B) A full-length model of *Homo sapiens* EEA1 is shown, colored by pLDDT (red – worst to blue – best). By dividing the full-length sequence into fragments (C), the resulting models are predicted with higher confidence (D), and can be analysed for local properties not seen in the full-length model (E).

CCfrag is implemented as a Python module that contains two main classes, the divider and the integrator. The divider is used to partition a full-length sequence according to a user-defined *specification*, which is defined by a window length, overlap length, and oligomeric state, and its output consists of the FASTA files necessary to run the AlphaFold predictions (Fig. 1C). After running AlphaFold predictions (Fig. 1D), the integrator module extracts a number of features from the models, and incorporates them into a rich per-residue representation that can be displayed graphically (Fig. 1E) or used for further analysis.

The divider module accepts various parameters, the most important of which are the window size (*L*), length of overlap between contiguous fragments (*O*), and oligomeric state (*N*). These define a specification, noted in the framework of the program as N_L_O (e.g. 2_30_15 would be 30-residue windows with 15-residue overlap, modeled as a dimer). Depending on these parameters, the module uses text string operations on the input full-length sequence to generate a set of queries appropriately formatted for AlphaFold. The minimum overlap size is 0 (no overlap), and the maximum is the length of the sequence minus one. If during the windowing of the input sequence, the C-terminal fragment is not long enough to produce a full window-sized fragment, the window size parameter (*L*) is given priority, and the overlap is increased for that last fragment. An additional feature of CCfrag is the possibility of setting a flanking sequence (flank), which will be attached N- and C-terminally to each window for the AlphaFold modeling, but will be removed in the integration step. The addition of a flanking sequence does not contribute in itself to the scores during integration (see below), but it can be used to promote the folding of the fragments; this feature is inspired by experimental techniques used to study coiled coils, whereby the addition of GCN4 adaptors is routinely used to promote folding and subsequent crystalization (Hernandez Alvarez *et al.* 2008). The output of the divider module consists of a parameter file (*parameters.json*), a table of constructs (*constructs.csv*), and a folder (*queries*) which contains the input FASTA files for AlphaFold.

CCfrag does not implement a wrapper to run AlphaFold. This is a necessary limitation given the variety of ways in which users can run AlphaFold (e.g. local machine, high performance cluster), but also a way to decouple the concept of fragment-based modeling from the prediction software itself. Thus, the program can be easily updated to work with newer and potentially better sequence-to-structure prediction programs. The examples shown in this article were run in ColabFold (Mirdita *et al.* 2022) version 1.5.2, using the *alphafold2_multimer_v3* model, with a maximum of five rounds of recycling, default MSA generation, and no templates. CCfrag also provides limited support for ESMfold (Lin *et al.* 2023), which offers a greatly increased prediction speed at the expense of lower accuracy (as well as not generating PAE scores, which are particularly useful to evaluate the robustness of oligomeric predictions). The generally poor performance of ESMfold (Supplementary Fig. S1) can be ameliorated by using the—*flank* option to add flanking coiled-coil sequences to promote the correct folding of the fragments (Supplementary Fig. S2).

After the AlphaFold models are generated, the integrator module reads most of its required parameters from the configuration file (*parameters.json*) and the list of constructs (*constructs.csv*) generated by the divider module. The user can specify which features will be extracted or computed from each window. CCfrag includes functions to extract pLDDT and PAE (the average), as well as a function to determine whether the models are parallel or antiparallel, based on the C-alpha inter-chain distances. The pLDDT (predicted local-distance difference test) is a per-residue measure of confidence in the accuracy of the predicted atomic coordinates (Jumper *et al.* 2021). It ranges from 0 to 100, where higher values indicate greater reliability, with scores above 90 suggesting near-atomic resolution. The PAE (predicted aligned error) is a matrix that measures the expected positional error between residue pairs in a predicted protein structure (Evans *et al.* 2022). For the detection of orientation, the program computes the distances between equivalent residues and compares their average to the same computation performed with the reversed residue index; if the reversal reduces the average distance, an antiparallel arrangement is inferred. CCfrag also includes a wrapper to run SOCKET (Kumar and Woolfson 2021) to detect knobs-into-holes interactions, the hallmark of coiled-coil structures. The addition of arbitrary features (e.g. solvent-accessible surface area) is possible, but requires defining a new function within the source code (an example is provided in the documentation). The integrator module computes the chosen feature-extraction functions for the AlphaFold models of all the fragments and stores each feature value alongside its corresponding position in the full-length sequence. The output of the integrator module is a table that stores per-residue values for each combination of feature and specification. During the integration process, the features of overlapping windows are flattened *via* averaging, although this can be customized. This means that, for the case of the parallel/antiparallel feature (numerically encoded as 1 and 0 respectively), some positions of the sequence may have a value of 0.5, meaning that half of the overlapping models showed a parallel arrangement, and the other half an antiparallel one; as illustrated in the next section, this can be interpreted as the lack of topological encoding in the local sequence.

## 3 Results

In this section, three examples of the use of CCfrag are presented. The examples focus on various long fibrous coiled-coil proteins, as these can be challenging to AlphaFold. A summary of the set of proteins used in the examples is provided in Supplementary Table S1. The code to generate and visualize these examples is included in the GitHub repository in the form of Jupyter notebooks.

### 3.1 Scanning long coiled coils for folding potential

**Figure 2.**
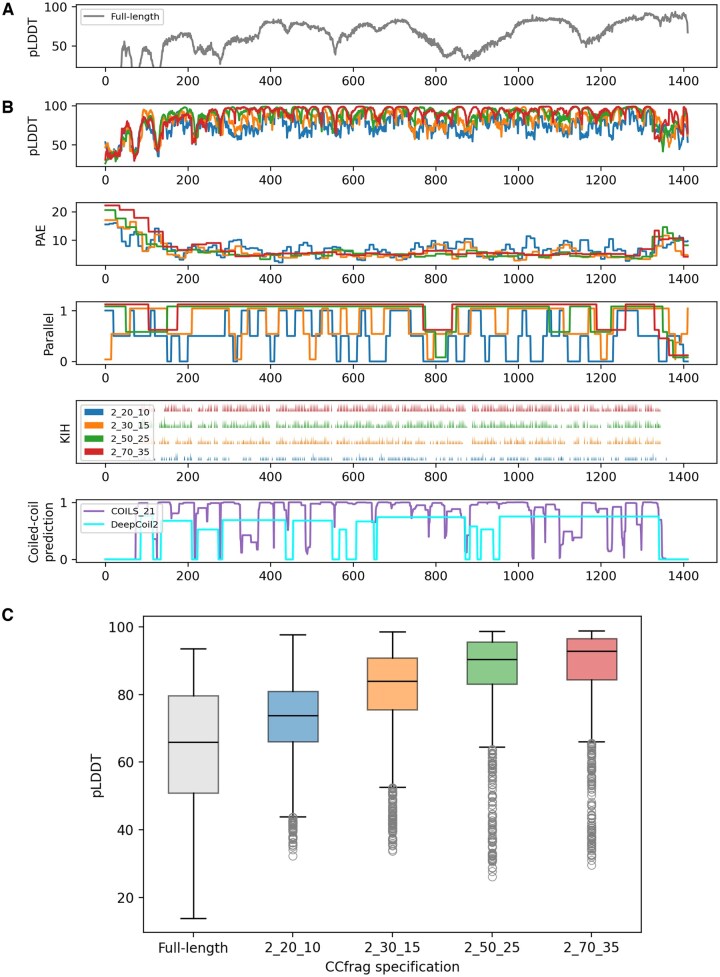
Comparison between full-length and piece-wise modeling of EEA1 from *Homo sapiens*. (A) pLDDT plot of the full-length dimer model. (B) Graphical summary of the CCfrag representation of the protein. It is modeled as a dimer in four different specifications with window sizes of 20, 30, 50, and 70 residues and an overlap of half the corresponding window size. Plots for various features are shown (pLDDT, PAE, bundle orientation, KIH interactions) for each specification (color-coded as in the legend in the left of the fourth panel). The features are averaged for each residue (since the overlap is half the window size, each residue is covered by two models, resulting in two values per-residue). In the bottom panel, coiled-coil prediction probabilities for COILS (window size = 21) and DeepCoil 2 are shown. (C) Boxplot representations for the pLDDT scores across different specifications, including the full-length model. A cartoon render of the full-length model is shown in Fig. 1B.

EEA1 (Early Endosome Antigen 1) is a protein that features an N-terminal zinc finger domain, a long parallel dimeric coiled coil, and a C-terminal FYVE domain. It has been extensively studied for its involvement in endosomal trafficking, where its coiled-coil domain is thought to switch between extended and flexible states (Murray *et al.* 2016). Using CCfrag to model EEA1 fragment-wise shows that the pLDDT scores are significantly better than those of the full-length prediction (Fig. 2A and C), with the largest window (*L* = 70) showing the best scores (Fig. 2B and C). On the other hand, it can be observed that shorter windows generally do not encode for a parallel orientation, except for some segments that seem to pull from their neighboring residues in larger window sizes (Fig. 2B); e.g. the 400th residue is found in an antiparallel orientation when modeled within a 20-residue context, but the neighboring segments promote the adoption of a parallel arrangement. As expected, there is significant overlap between the SOCKET knobs-into-holes (KIH) detection and the sequence-based coiled-coil predictors DeepCoil2 (Ludwiczak *et al.* 2019) and COILS (Lupas *et al.* 1991). Notably, the absence of KIH interactions also coincides with weak coiled-coil predictions in one or the other program, suggesting that these correspond to flexible regions or segments with ambiguous topological specificity.

We observe a similar behavior in Myosin, a motor protein responsible for muscle contraction and various cellular movements. While the full-length model of its coiled-coil stalk shows several breaks with poor pLDDT values, the fragment-based models suggest a continuous fiber (Supplementary Fig. S3), whose confidence scores improve with the size of the fragment. Notably, this also illustrates that the improvement in scores associated with the windowing procedure is only found in the coiled-coil region, and not the N-terminal ATPase domain. This is likely related to the highly-local contact maps that are typical of parallel coiled-coil fibers.

### 3.2 Scanning for non-canonical coiled coils

In recent work, we described a number of protein families that predominantly featured hendecad coiled-coil repeats (Martinez-Goikoetxea and Lupas 2023), and pointed out that these are a particularly difficult targets for AlphaFold. These non-canonical coiled coils are underrepresented in natural proteomes, and possibly as a result, are poorly detected by sequence-based coiled-coil predictors. Using CCfrag to model a member of the MACH family illustrates how piece-wise modeling can be applied to essentially scan sequences for potential KIH interactions, thus detecting coiled coils that even coiled-coil specific methods fail to predict (Supplementary Fig. S4).

A better studied non-canonical coiled-coil stalk is found in the surface layer protein tetrabrachion from the archaebacterium *Staphylotermus marinus*. Although it is also poorly detected by sequence-based coiled-coil prediction methods, scanning the sequence piece-wise with AlphaFold can be used to detect the presence of the coiled-coil stalk (Supplementary Fig. S5). Notably, the predicted fragments confirm previous analyses of the sequence (Peters *et al.* 1996), in terms of the annotation of different coiled-coil periodicities.

### 3.3 Multi-state modeling

**Figure 3.**
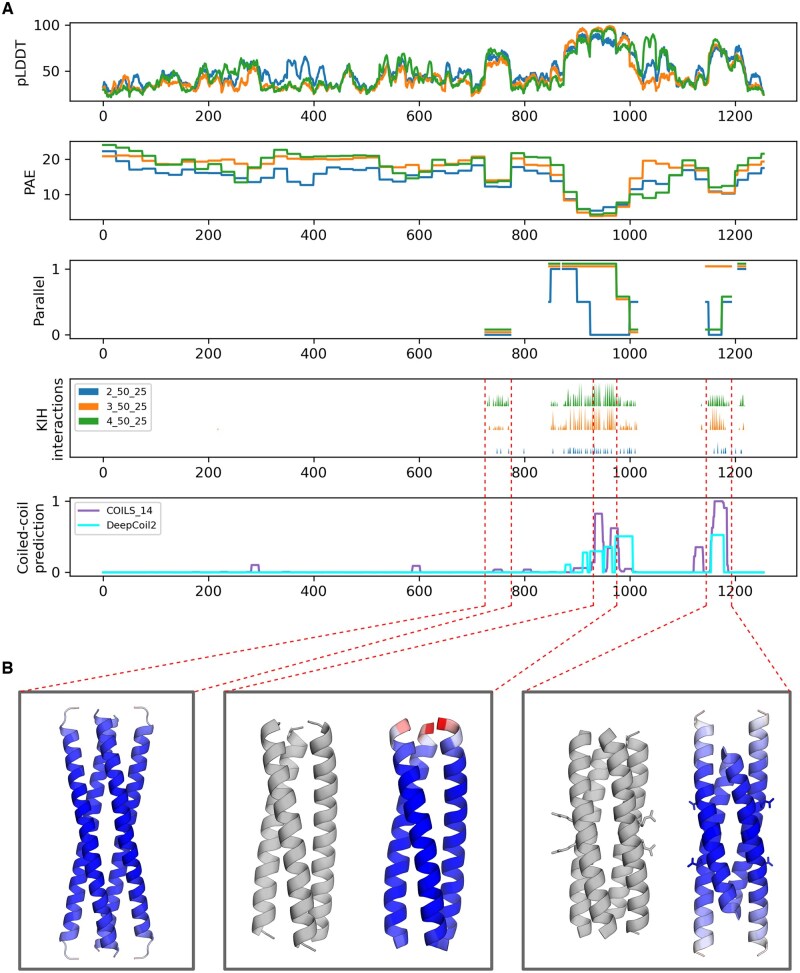
Graphical summary of the CCfrag representation of the spike protein of human SARS coronavirus (UniProt P59594). The spike protein is involved in the fusion between the viral and cellular membranes, and although it is not a fibrous protein, it features two functionally relevant coiled-coil domains, known as HR1 and HR2 (for heptad repeat). (A) The protein is modeled as a dimer, a trimer, and a tetramer, each in 50-residue windows with a 25-residue overlap. There are three regions which are more confidently predicted than their surrounding context (high pLDDT, low PAE), two of which correspond to HR1 and HR2. (B) (Left) Fragment 725–775 of the protein is predicted as an interlocking array of coiled coils. (Center) Fragment 925–975 is predicted as a parallel trimer, nearly identical to the experimentally solved structure (1ZVB, in grey; RMSD: 0.4 Angstroms). (Right) Fragment 1150–1200 is predicted as an antiparallel tetramer, although with a register different of that of the experimentally solved structure (PDB 1ZV7, in grey). The predicted structures are colored by pLDDT (red – worst to blue – best).

It has been shown that AlphaFold prediction quality metrics such as pLDDT and PAE can inform of the likely oligomeric states of protein complexes (Madaj *et al.* 2024, Schweke *et al.* 2024). In the context of fragment-based modeling of coiled coils, oligomeric state prediction is particularly challenging due to the fact that coiled-coil sequences can often assemble into various nearly-isoenergetic oligomeric states (Harbury *et al.* 1993). A consequence of this is that when coiled-coil fragments are taken out of their native context, they often crystallize in oligomeric states that do not correspond to the native stoichiometry of the full-length protein. Nevertheless, these fragments inform of the topological specificity that is encoded locally. A great example of this can be found in the spike protein of SARS Coronavirus, whose coiled-coil segments have been crystallized as trimers and tetramers (Deng *et al.* 2006), even though the full-length protein is known to assemble into a trimer. Using CCfrag, we modeled this protein as dimers, trimers, and tetramers, and observed that some of the predicted models matched experimentally-validated coiled-coil domains present in the spike protein (Fig. 3). We also observed additional fragments that, even though they do not form coiled coils, were nevertheless predicted as coiled-coil structures. This suggests these fragments feature cryptic coiled-coil folding potentials, and that could fold as such if removed from their native context.

### 3.4 Conclusions

CCfrag extends the functionality of AlphaFold by facilitating the process of dividing a protein sequence into smaller overlapping fragments, running AlphaFold predictions on these, and integrating the resulting models into a rich per-residue representation. We find that this piece-wise modeling can improve the robustness of the predicted models in the case of long coiled coils, even non-canonical ones. We hypothesize that this is due to the lack of representation of such structures in the Protein Data Bank, from which AlphaFold was trained. It is possible that during the AlphaFold model generation, this biased representation causes models with long extended helices to spuriously bend in order to increase the number of contacts, to more closely resemble the training set. Running predictions on windowed segments seems to largely avoid this issue, while also generally improving confidence scores.

Additionally, we propose that this representation can reveal local features not seen in full-sequence models, such as oligomerization preference or folding propensity. It is of particular interest the case of long, parallel fibers, such as EEA1 or Myosin, which show interruptions in the stalks of their full-length models, yet show as continuous coiled coils when modeled piece-wise. This could be indicative of the presence of alternative potential conformations, which would likely be relevant for the function of these proteins, e.g. as part of their activity modulation (Yang *et al.* 2020). We think that CCfrag will be specially useful in the study of long, parallel dimeric-, trimeric-, and tetrameric-coiled-coil fibers, where fragment-based modeling works best.

## Supplementary Material

vbae195_Supplementary_Data

## Data Availability

CCfrag, together with its documentation and additional examples, is available at https://github.com/Mikel-MG/CCfrag.
