# Supplementary material for: CCfrag: scanning folding potential of coiled-coil fragments with AlphaFold
Source: Bioinform Adv. 2024 Dec 6;5(1):vbae195. doi: 10.1093/bioadv/vbae195 (PMC11676326; doi:10.1093/bioadv/vbae195)
Supplement: vbae195_Supplementary_Data [file vbae195_supplementary_data.pdf]

Supplementary Material

CCfrag: Scanning folding potential of coiled-coil fragments with AlphaFold

Mikel Martinez-Goikoetxea
Department of Protein Evolution, Max Planck Institute for Biology, 72076 Tübingen, Germany
E-mail: mikel.martinez@tuebingen.mpg.de

Protein	Sequence ID	Description
EEA1	UniProt Q15075	Long, dimeric canonical coiled-coil stalk
Myosin	PDB 6XE9_A	Long, dimeric canonical coiled-coil stalk
MACH	NCBI WP_132310275	Long, trimeric hendecad coiled-coil stalk
Tetrabrachion	NCBI AAC44118.1	Long, tetrameric heptad and hendecad coiled-coil stalk
Coronavirus spike protein	UniProt P59594	Short coiled-coil segments within an otherwise non-fibrous trimeric protein

Table S1. Summary of the proteins discussed in the main manuscript.

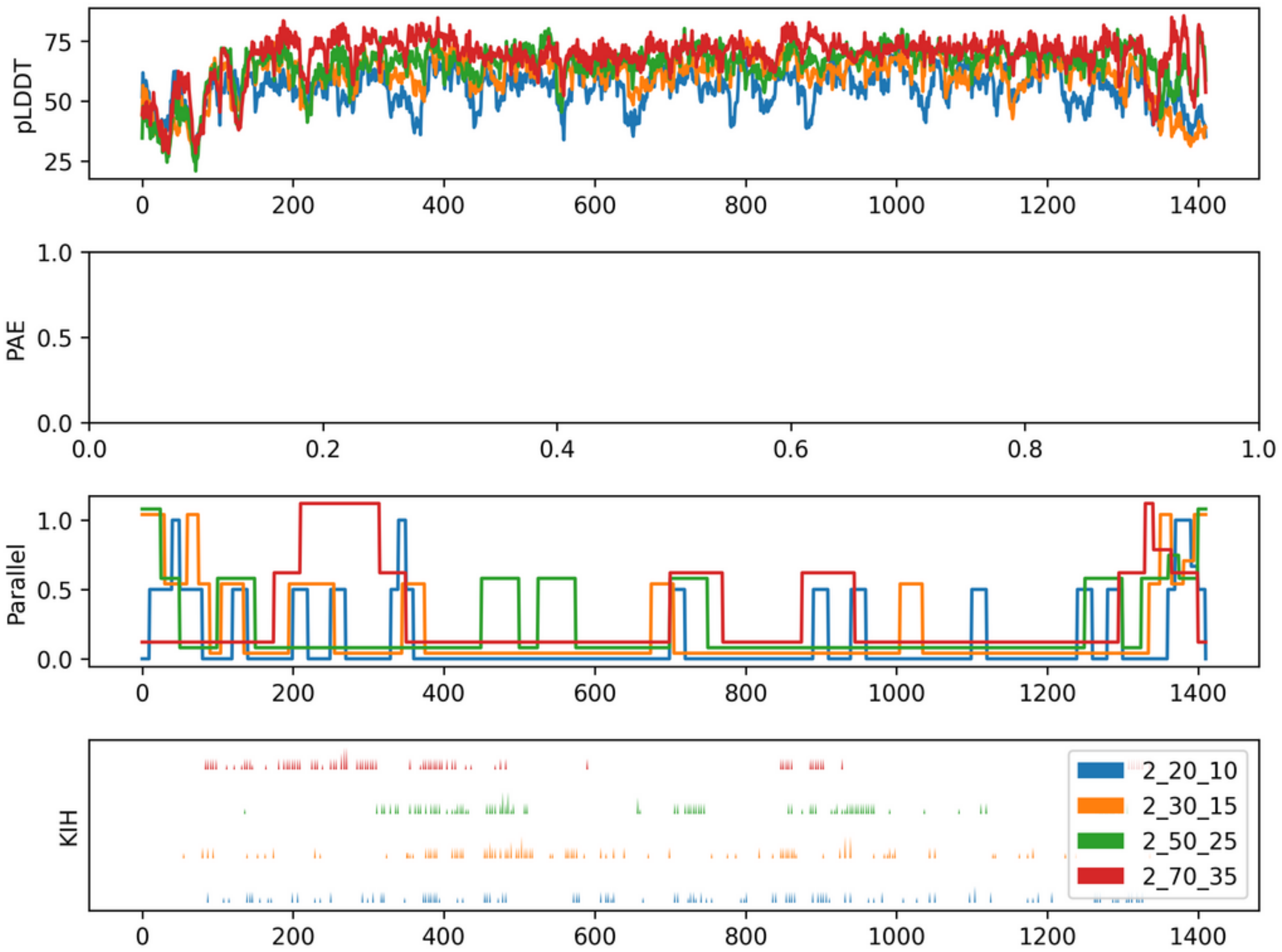

Figure S1. Graphical summary of the CCfrag representation of EEA1 from *H. sapiens*, modeled with ESMfold. Most of the fragments are predicted as antiparallel assemblies, and few of them feature the expected knobs-into-holes interactions, typical of coiled coils.

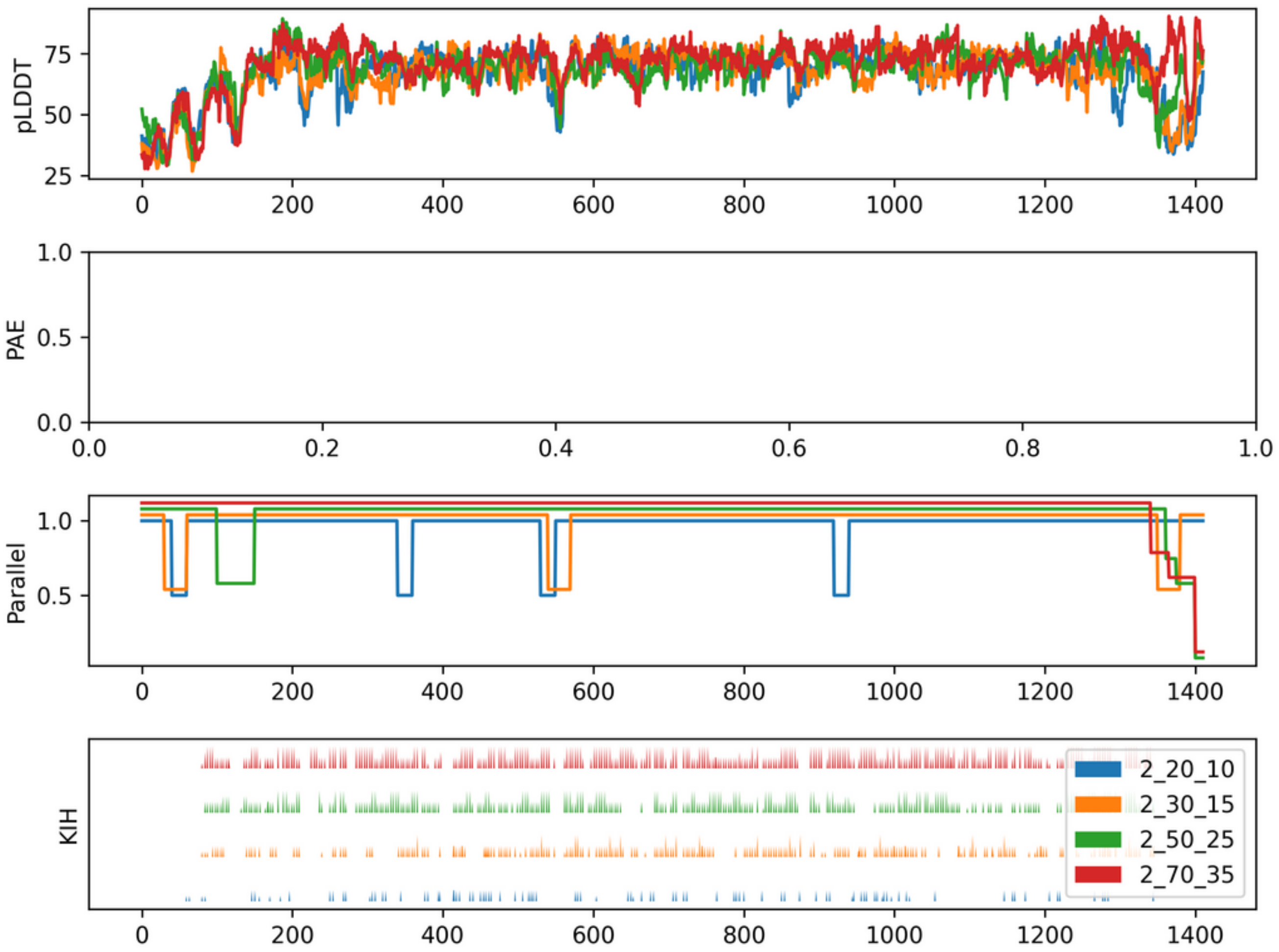

Figure S2. Graphical summary of the CCfrag representation of EEA1 from *H. sapiens*, modeled with ESMfold, but adding flanking coiled-coil sequences (GCN4) to promote the correct folding. As opposed to Fig. S1, the fragments are largely predicted as parallel assemblies, and most of them feature the expected knobs-into-holes interactions, typical of coiled coils.

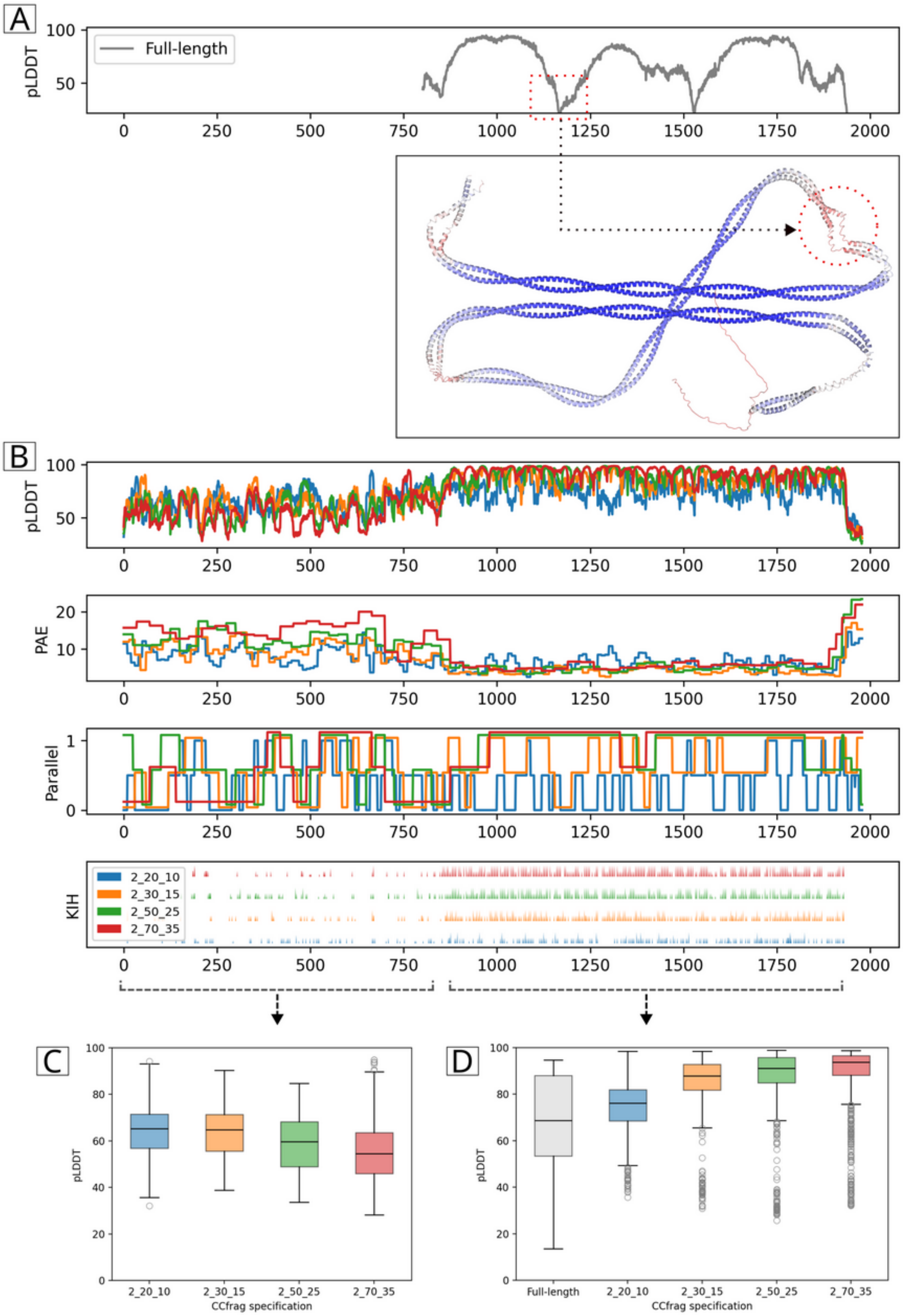

Figure S3. Comparison between the full-length model of the Myosin coiled-coil stalk and its CCfrag representation. (A) PyMOL render and pLDDT score plot of the full-length model of the Myosin coiled-coil stalk. The PyMOL render has been colored by pLDDT, from worst-red to best-blue. (B)

Graphical summary of the CCfrag representation, with plots of pLDDT, PAE, bundle orientation, and knobs-into-holes (KIH) interactions for every specification. Only the coiled-coil stalk segment shows KIH interactions. (C) Boxplot representation of the pLDDT scores for residues (C) outside and (D) within the coiled-coil stalk. Only for residues within the coiled-coil stalk (D) do the pLDDT scores improve with the size of the fragment.

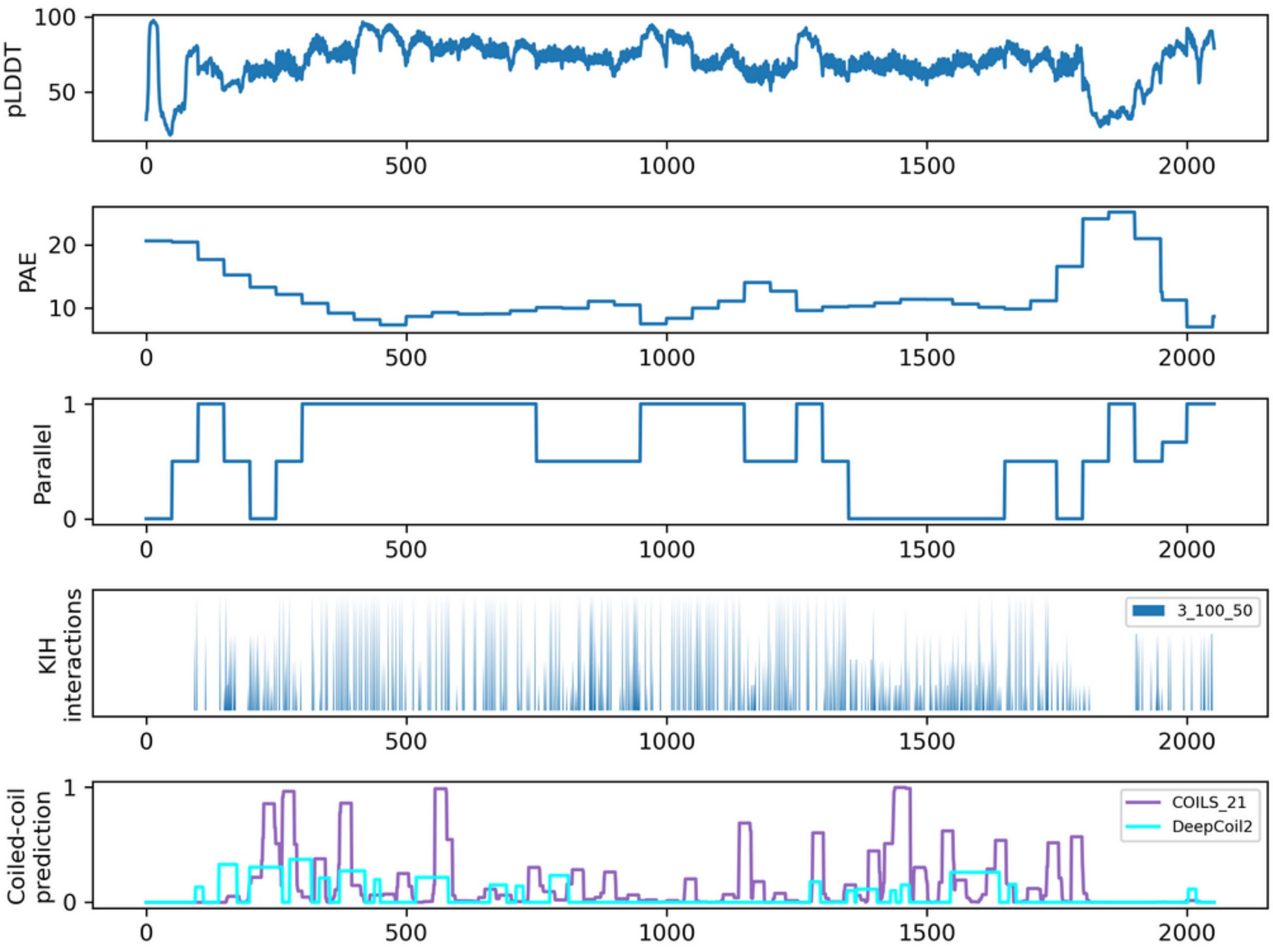

Figure S4 . Graphical summary of the CCfrag representation of WP_132310275 from *Martelella mediterranea*, a member of the MACH protein family; according to interactive sequence analyses, these proteins feature an extensive hendecad coiled-coil domain. The protein is modeled as a trimer, in a specification of 100-residue windows with 50-residue overlap. Note that even though the sequence-based coiled-coil prediction is very poor, the knobs-into-holes (KIH) interactions can be detected in the structural models along subsequent fragments, supporting the presence of a long coiled-coil stalk.

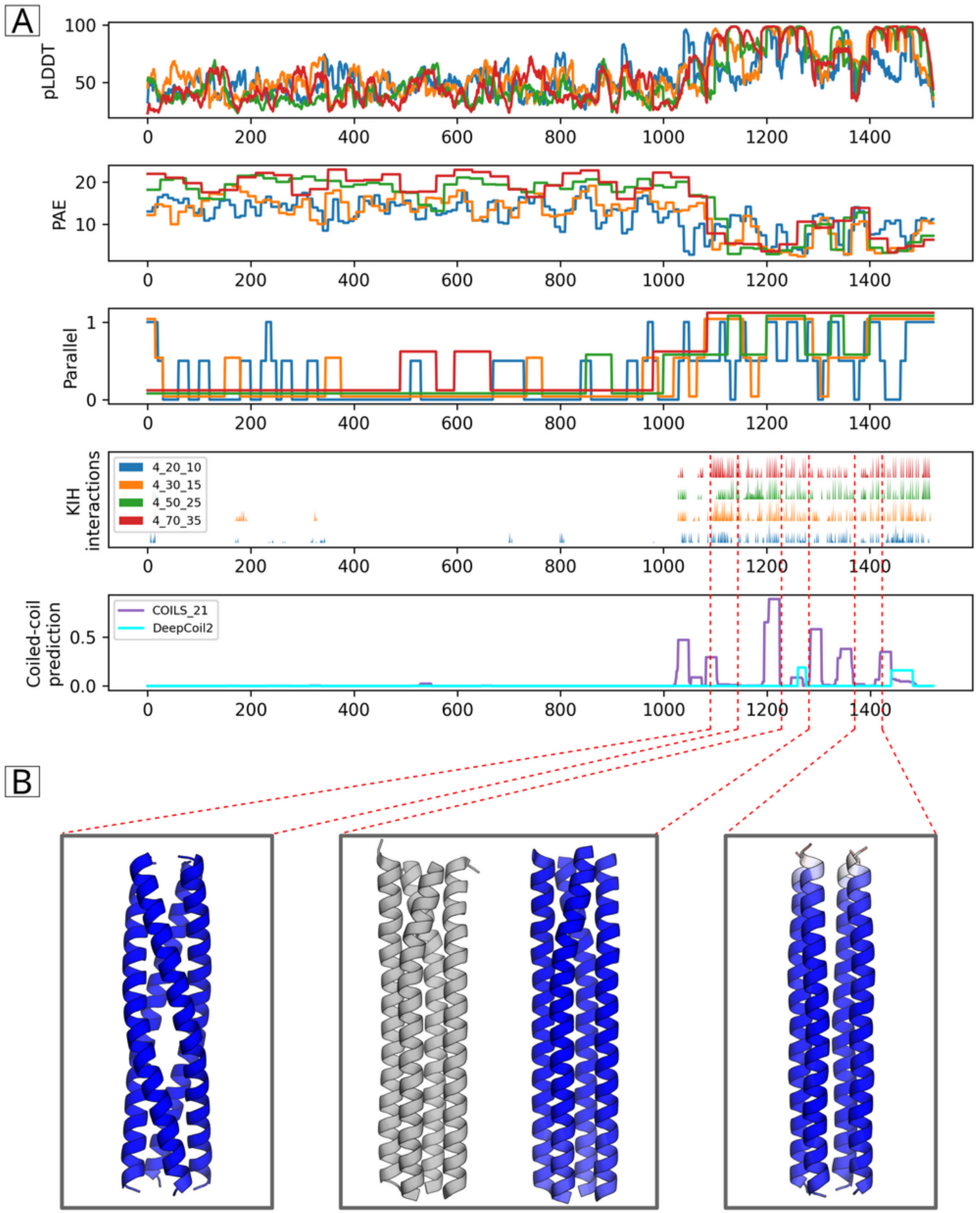

A
pLDDT
PAE
Parallel
KIH interactions
4_20_10
4_30_15
4_50_25
4_70_35
Coiled-coil prediction
COILS_21
DeepCoil2
B

Figure S5. Graphical summary of the CCfrag representation of the surface layer protein Tetrabrachion from *Staphylothermus marinus*. Its coiled-coil stalk features extended segments of non-canonical coiled coils which cannot be effectively predicted from sequence. (A) The protein is modeled as a tetramer, in windows of 20, 30, 50, and 70 residues, and an overlap of half the window size. Plots of pLDDT, PAE, bundle orientation, and knobs-into-holes (KIH) interactions are shown for every specification, as well as the poor sequence-based coiled-coil prediction. (B) Selected fragments are shown as PyMOL renders to illustrate the change in bundle periodicity (supercoiling) along the stalk. Left) part of the heptad stalk with its characteristic left-handed supercoiling; Center) comparison with the only experimentally-solved fragment (1FE6, in grey; RMSD: 0.45 Angstroms); Right) part of the hendecad stalk, which features essentially straight helices. The predicted structures are colored by pLDDT (red-worst to blue-best).
